# Relationship between cognitive performance and neurometabolite levels in the prefrontal and medial temporal cortex from childhood to adulthood

**DOI:** 10.1192/j.eurpsy.2026.11628

**Published:** 2026-07-31

**Authors:** S. A. Gutiérrez de Larraya i Casado, M. Ortuño, A. Fortea, E. Muñoz-Moreno, R. Borràs, I. Martínez-Serrano, M. Masias, P. Camprodon-Boadas, E. de la Serna, I. Baeza, J. Castro-Fornieles, G. Sugranyes

**Affiliations:** 1 Department of Child and Adolescent Psychiatry and Psychology, Institute of Neuroscience; 2 Department of Psychiatry and Psychology, Institute of Neuroscience, Hospital Clínic de Barcelona; 3 Fundació de Recerca Clínic Barcelona-Institut d’Investigacions Biomèdiques August Pi i Sunyer; 4 Faculty of Medicine and Health Sciences, Neurosciences Institute, University of Barcelona, Barcelona; 5 Centro de Investigación Biomédica en Red de Salud Mental, Madrid; 6 Experimental 7T MRI Unit, Magnetic Resonance Imaging Core Facility; 7 Cardiovascular Institute, Fundació de Recerca Clínic Barcelona-Institut d’Investigacions Biomèdiques August Pi i Sunyer; 8 BCN-MedTech, Department of Information and Communication Technologies, Universitat Pompeu Fabra, Barcelona, Spain

## Abstract

**Introduction:**

Several studies have evaluated neurometabolic correlates of cognitive abnormalities in psychiatric disorders using magnetic resonance spectroscopy (MRS). There is little evidence on the metabolite-cognition relation, particularly in childhood and adolescence, period when many psychiatric disorders begin.

**Objectives:**

Our aim is to assess the relation between 4 cognitive functions and levels of neurometabolites in the dorsomedial prefrontal cortex (dmPFC) and the medial temporal lobe (mTL) regions in a sample of healthy child and adolescents.

**Methods:**

83 healthy subjects (aged 7.6-27.2 years-old, mean age 16.7±4.3) were recruited, each one underwent the acquisition of two magnetic resonance imaging (MRI) sequences on a 3T scanner: a T1-weighted sequence and a MRS in two volumes of interest (VOIs) located in the dmPFC (2x2x2 cm³) and the mTL (3x2x2 cm³). MRS data was processed in order to obtain absolute ratios of glutamate, glutamate+glutamine (Glx), myo-inositol, glycerophosphocholine+phosphocholine (tCho), N-acetylaspartate+N-acetylaspartylglutamate (tNAA) and creatinine+phosphocreatine (tCr). Cognition was assessed employing several cognitive tests: intellectual quotient (IQ) and working memory (WM) specified for Wechsler Intelligence Scale for Children (WISC-IV) or Wechsler Adult Intelligence Scale (WAIS-IV) -depending on age-; sustained attention (SA) and executive function (EF) specified for Conners’ Performance Test (CTP) and Wisconsin Card Sorting Test (WCST). Linear models were conducted with each cognitive function as dependent variable, each metabolite as independent, and sex and age as covariates. False discovery rate (FDR) multiple comparisons correction was employed to identify the most significant findings.

**Results:**

Inspection of residuals confirmed that the concentration for each of the studied metabolites followed a linear trajectory. In the dmPFC, there was a significant negative relation between Glx and SA (beta=2.22, SE=0.78, t=-2.86, p=0.006), that remained significant after multiple comparisons corrections (pFDR=0.036). Also, the relationship between executive function and tCR in dmPFC were found primarily significant (p=0.048), however this did not survived multiple comparisons corrections (pFDR=0.277). In the mTL, there was a significant relation between IQ and tCho (beta=-15.56, SE=7.50, t=-2.08, p=0.043), between SA and tCho (beta=9.11, SE=4.40, t=2.07, p=0.044) and between EF and tNAA (beta=5.15, SE=1.90, t=2.70, p=0.012), but these didn’t remained significant after multiple comparisons corrections (pFDR=0.26, 0.27 and 0.071, respectively).

**Conclusions:**

The relation detected between SA and Glx in the prefrontal cortex extends previous findings in adult samples. This helps further our understanding of the relationship between neurometabolites and cognitive domains at this key age of neurodevelopment.

**Disclosure of Interest:**

None Declared

